# Biologically Aware Lighting for Newborn Intensive Care

**DOI:** 10.21203/rs.3.rs-3120637/v1

**Published:** 2023-07-10

**Authors:** James Greenberg, Katherine Gruner, Lousette Rodney, Jaime Struve, Daniel Kang, Yuying Cao, Richard Lang

**Affiliations:** Cincinnati Children’s Hospital Medical Center; Cincinnati Children’s Hospital Medical Center; Cincinnati Children’s Hospital Medical Center; Cincinnati Children’s Hospital Medical Center; Cincinnati Children’s Hospital Medical Center; Cincinnati Children’s Hospital Medical Center; Cincinnati Children’s Hospital Medical Center

## Abstract

**Objective:**

We designed and implemented a novel neonatal intensive care (NICU) lighting system to support current understanding of sunlight-coupled physiology.

**Methods:**

We created a system that generates wavelengths corresponding to the known blue and violet activation spectra of non-visual opsins. These are known to mediate energy management and related physiologic activity.

**Results:**

Light produced by the system spans the visible spectrum, including violet wavelengths that are blocked by modern glazing and not emitted by standard LED fixtures. System features include automated light and dark phases that mimic dawn/dusk. The system also matches length of day seasonality. Spectral composition can be varied to support translational research protocols. Implementation required a comprehensive strategy to inform bedside providers about the value and use of the lighting system.

**Conclusion:**

Full-spectrum lighting for the NICU is feasible and will inform optimization of the NICU environment of care to support optimal neonatal growth and development.

## Introduction

The most dynamic phases of growth, metabolic activity, and organ development during the human lifespan begin with the fetal period and continue through the early years of extrauterine life. There is much evidence that this crucial period of development is profoundly impacted by a myriad of environmental stimuli such as sound and light. For example, the plasticity of the auditory cortex in preterm infants is preferentially responsive to intra-uterine maternal sounds (1). Another example emerges from studies of circadian stimuli in neonates. The onset of cyclical patterns is detectable during the early second trimester and become more intense as term gestation approaches (2). Clinical studies of basic cycled lighting environments point to a positive impact on weight gain, growth, and length of stay (3); (4); (5) when compared with conventional lighting.

Environmental stimuli experienced by hospitalized infants in the NICU are very different from those within the intrauterine setting. When a birth is preterm, these contrasts are exacerbated by early termination of environmental inputs provided through the mother. The NICU setting confronts the newborn with a litany of unnatural stimuli ranging from equipment alarms to artificial lighting.

As we consider the impact of the environment on early growth and development, we must include a growing body of new knowledge regarding the biology of light sensing. Beyond cycled (light/dark) lighting, environmental light exerts specific biological effects through light sensitive G-protein coupled receptors, known collectively as opsins (Table 1). In humans, these respond to wavelength ranges within the 380–700 nm range of the visible spectrum (6); (7); (8). Of particular interest, three opsins detect light but do not directly contribute to vision. OPN4 (melanopsin) has generated substantial investigation following its confirmed functional role in circadian entrainment influencing sleep/wake cycles (9). The functions of two additional nonvisual opsins, OPN3 (encephalopsin) and OPN5 (neuropsin) are now understood to impact fundamental physiologic and metabolic processes including temperature regulation, energy management, and growth (10); (11). These findings have led to the recognition that mammalian physiology is intimately coupled to the sunlight cycle and requires interaction with wavelengths not produced by standard artificial lighting sources. As knowledge about these evolutionarily conserved nonvisual opsins expands, it is highly plausible that their activation is necessary for optimal NICU outcomes (12). To address this idea, we generated a NICU lighting system capable of satisfying the needs of sunlight coupled physiology (SCP).

The construction of a new Critical Care Building (CCB) at Cincinnati Children’s Hospital Medical Center (CCHMC) offered a unique opportunity to incorporate SCP lighting into clinical care settings. We chose the level IV NICU as the installation site in the CCB given the relatively long average length of stay (approximately 35 days) and the very dynamic growth and development intrinsic to this patient population. Here we detail the design and deployment of that lighting system and how we will use this system to advance understanding and improve outcomes for the NICU patient population.

## Methods

### Design Specifications:

We sought to create a NICU lighting system that satisfies established, design specifications presented in detail by White RD *et al* (13). We also incorporated relevant general design and architectural standards such as those published by the Illuminating Engineering Society (IES). We envisioned this lighting system not only as standard patient room lighting solution, but also as a device to support circadian and opsin-based translational research. Within this conceptual framework, we defined four fundamental performance characteristics:

1. Automated diurnal light cycling to reflect day-night cycles,
2. Generation of light wavelengths for activation of visual and non-visual opsins (Table 1),
3. Dynamic spectral variation corresponding to daily sunrise-midday-sunset progression,
4. Length of day seasonal variation.

Our concept required a control system that supported the functionality noted above and allowed for variation in spectral power distribution, light-dark cycles, and seasonality. We also required a lighting control system that could be easily managed by bedside care providers with minimal disruption to patient care activities. To support the functionality required for NICU patient care operations, the luminaire generates high-quality broad-spectrum white light at a 3500K color temperature consistent with standard lighting specifications at Cincinnati Children’s. The lighting generates a cyanosis observation index threshold of <3.3 to support accurate patient observations based on Australia/New Zealand standard AS/NZS 1680.2.5:2018.

### Daylight and artificial light spectral analysis:

Daylight, defined as the sum of direct and indirect solar irradiance was collected through a calibrated spectrometer with peak sensitivity range of 350–800 nm (STS-VIS, Ocean Insight, Orlando, FL). We mounted the spectrometer on a mast located 25 feet above the roofline of the 14 story Cincinnati Children’s Clinical Sciences Pavilion located about 500 feet from the CCB. The location allows for 97.5% coverage from horizon to zenith in all directions. Minimal shadowing (2.5%) is due to an air terminal for lightning protection located a few feet north of the spectrometer detector. The detector is a 1024-pixel CMOS device equipped with cosine corrector housed in a weather protection dome and connected to the spectrometer via fiberoptic cable. Data was collected every 10 minutes and downloaded to data acquisition software. In addition, we obtained spectral measurements of the standard ambient lighting found in the former CCHMC NICU (“old NICU,” closed in November 2021) and the CCB NICU (“new NICU”). These measurements employed a custom-built spectrometer designed and manufactured by Ocean Insight. This device contained four STS-VIS spectrometers to capture irradiance from a variety of locations within the NICU environment including open cribs, radiant warmers, and isolettes (with and without coverings). Additional details regarding the lighting detection systems are provided in Cao *et al*. (submitted for publication).

### Luminaire Manufacture and Installation:

Once all spectral and luminaire design parameters were defined, we assembled a multidisciplinary manufacturing team to create working luminaire prototypes and develop a control interface that could support various user needs ranging from bedside care provider to researchers. Each working wall-mounted prototype was evaluated in-person by the design and research teams in a full-scale model patient room. Parameters included extensive measurements of spectral properties and operation of the control interface. Patient room installation required coordination with the CCB construction manager, subcontractors, and the Cincinnati Children’s information technology, physical plant, and clinical engineering departments. The prototype evaluation phase documented the need for a portable version of the lighting system to address potential areas of limited lighting coverage with certain patient settings and the possibility of research settings outside the CCHMC NICU. The portable fixture meets the same lighting specifications as the wall-mounted fixture.

## Results

### Daylight and NICU spectral analysis (Figure 1):

Our outdoor spectrometer measurements detected a comprehensive span of wavelengths between 350–900 nm. As expected, the intensity of photonic flux rises and falls corresponding to transitions from sunrise to midday to sunset. Key wavelengths corresponding to nonvisual opsin activation vary throughout daylight progression. Violet and blue values dominate at sunrise and sunset. Artificial lighting conditions in the old and new NICU (without the spectral lighting source) generated spectra of limited wavelength coverage consistent with known characteristics of artificial light sources. Notably, both old and new NICU (without the spectral lighting source) environments lacked wavelengths in the violet range.

### Luminaire Design and Performance (Figure 2):

The spectral lighting system was installed in 48 patient rooms in the CCB and complements additional standard LED fixtures that are incorporated in all CCB patient rooms. The spectral lighting system luminaire includes a 6 LED light engine capable of generating all wavelengths present in natural daylight with an emphasis on visual and nonvisual opsin activation. Separate controls are provided for each LED channel allowing for variation in the spectral composition to emulate the sunrise-midday-sunset progression. The spectral output of each NICU luminaire can be modulated to support clinical research protocols to interrogate opsin function. For example, wavelengths corresponding to OPN5 activation can be omitted from the standard daytime progression to mimic standard artificial lighting conditions. Similarly, length of day can follow seasonal variation or remain static. The system software supports a lighting condition archive. Researchers can assign lighting protocols for each patient room by remote access. The in-room control interface allows a bedside provider to assume control of the lighting system at any time if necessary for patient care.

### Clinical Practice:

**Figure 3:**
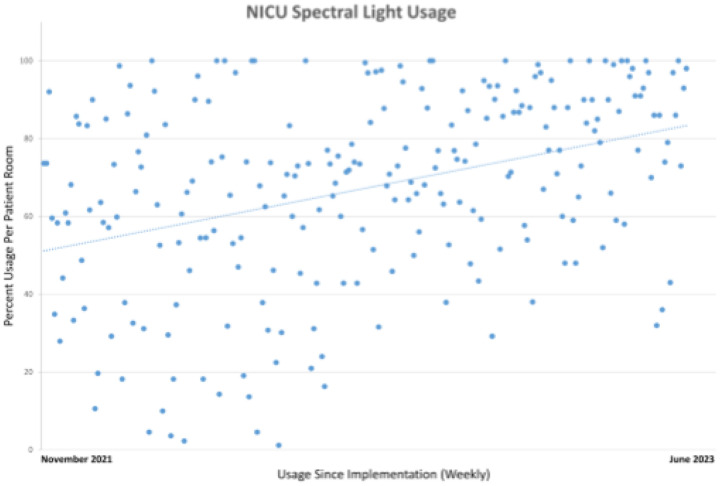
Weekly usage of the spectral lighting system was calculated from November 2021 to June 2023) for three randomly selected rooms. For each room, the percentage of spectral light usage was determined over the course of each week and recorded as a single data point. A progressive increase in use is shown over the measurement period.

The transition from the old NICU to the new CCB NICU brought dramatic changes in patient room configuration. In addition to the spectral lighting system, the new NICU rooms also included standard LED fixtures that generated an order of magnitude increase in light levels compared to the old NICU environment. Consequently, providers found the lighting environment esthetics bright in contrast to the old NICU. Providers raised concerns regarding patient sleep, exacerbation of certain pathophysiologic conditions such as pulmonary hypertension, and family acceptance. In Table 2, these are summarized along with approaches for support of patients, families, and staff. We incorporated our findings into a new NICU clinical practice guideline issued 6 months after the CCB patient move. Continuing education, staff presentations, and informal rounding supported adoption of the guideline and increased utilization of the spectral lighting (Figure 3).

### Clinical studies:

The design of the NICU lighting system supports research in a real-world clinical setting. Our current understanding of opsin biology points to their importance for energy management and, therefore, their potential impact on growth and development. While the past 25 years has seen a dramatic improvement in our ability to provide nutritional support for the NICU population, many important questions remain outstanding. For example, protein and energy intake predict brain growth. However, optimization of neurodevelopmental outcomes through nutritional management remains a challenge (14). Appropriate stimulation of OPN3 and OPN5 may play an important role and now can be interrogated through our NICU lighting system. Similarly, development of normal sleep/wake patterns are likely impacted by access to circadian signals that go beyond simple light/dark cycles.

## Discussion

We present the first lighting system to be designed to accommodate the new knowledge of sunlight coupled physiology. To our knowledge, this is the only cycled lighting system that generates wavelengths capable of OPN5 activation. This allows careful interrogation of the activity of OPN5 in a clinical setting. While the NICU is a compelling place for this initial phase of translational investigation, many opportunities exist in other locations such as long-term behavioral health facilities and other sites for management of chronic conditions. A growing body of evidence directly implicates OPN5 and violet light exposure to the progression of myopia and retinopathy of prematurity (15); (16). There is also substantial interest in the impact of the cycled lighting environment for providers and families. The ability to move from generic light-dark cycling to dynamic full-spectrum lighting that mimics natural variation in spectral composition from sunrise to sunset represents an important advance.

It is important to distinguish the spectral capacity of the NICU lighting system from lighting that varies color temperature over the course of a daytime period. Color temperature measures visual perception of light rather than actual spectral distribution. For example, the perception of violet can be created without the generation of wavelengths in the violet spectrum. Without violet photons, OPN5 stimulation will not occur.

At this early stage of translational research there are many unanswered questions. The “dose” of light needed to activate OPN5 is not precisely defined. Preclinical studies demonstrate that sufficient photons reach deep brain structures in mice to stimulate OPN5 (11). Similarly, a growing body of studies document activation of OPN3 and OPN5 in a variety of non-ocular locations such as skin and adipose tissue (10); (17). Opsins demonstrate absorption spectra over a range of wavelengths. For example, OPN5 can be stimulated at different efficiencies from 340–420 nm. Correlation of absorption spectrum to biological effect in a real-world clinical setting will require additional study.

Our growing understanding of OPN3 and OPN5 implies their fundamental role in metabolic activities and energy homeostasis. Many conditions encountered in the NICU are associated with growth failure such as gastroschisis, intestinal failure/short bowel, and bronchopulmonary dysplasia. The significance of body composition for neurodevelopmental outcome is an area of increasing interest for the NICU population. Circadian influences on energy management are likely to be very relevant to common NICU practices such as enteral feeding protocols and parenteral nutrition. For example, time of day and exposure to relevant daylight wavelengths may profoundly influence how a neonate utilizes macronutrients and energy substrate. Post-discharge outcomes, such as visual acuity and neurobehavior, may also be impacted.

As we consider environmental light exposure in the context of neonatology practice, many questions remain. The dose of daylight, including duration of exposure, intensity, and key wavelengths will require further refinement. This is particularly interesting when deep tissue structures expressing light-sensing opsins are considered. Electromagnetic wavelengths outside of the visual spectrum (infrared and ultraviolet) are also known to influence certain biologic processes through non-opsin mediators. Our focus here is restricted to wavelengths within the visual spectrum known to activate human opsins. Finally, energy management, utilization of macronutrients, and growth are fundamental to survival. It is reasonable to expect that pathways regulated through opsin stimulation are relevant to human growth and development. Our NICU lighting system will now allow interrogation of these pathways, supporting the further optimization of growth and development during a crucial stage of life.

## Figures and Tables

**Figure 1:**
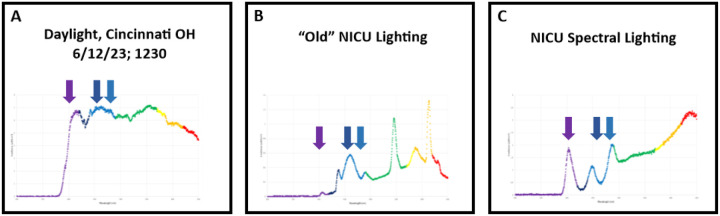
Spectral distributions were measured with a ST-UV-100 microspectrometer (Ocean Insight, Orlando, FL) calibrated to the DH-3P-BAL-CAL light source. Outdoor measurements (panel A) were taken with an integration time of 100μs to prevent oversaturation. For indoor room measurements, the cosine corrector was placed at the level of the patient bed with an integration time of 100 ms. The spectral distribution of ambient lighting conditions in the University of Cincinnati Hospital (“Old”) NICU is shown in panel B. Panel C depicts the spectral distribution of ambient lighting conditions in the Critical care Building NICU at midday. The arrows in each panel mark the approximate peak absorption of OPN5, OPN3, and OPN4 (left to right). Note the presence of violet photons in panels A and C.

**Figure 2:**
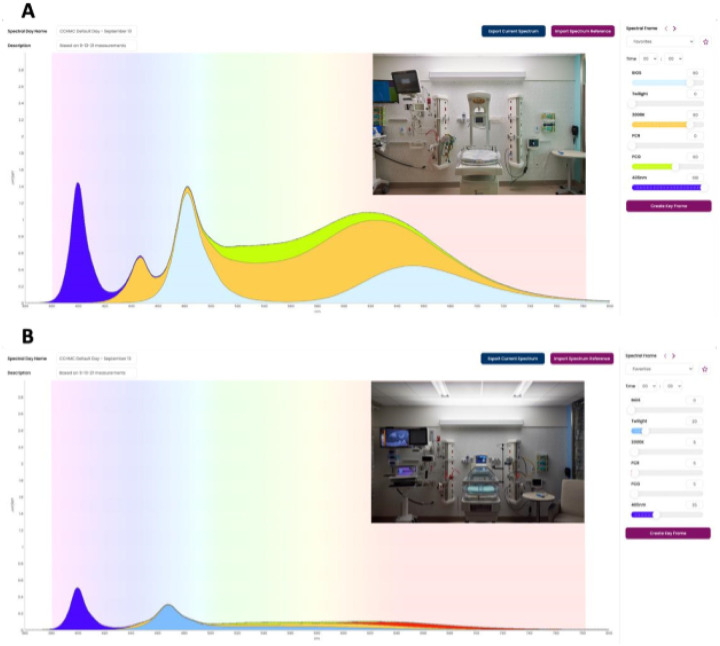
Spectral distribution recipes for light generated in the Critical care Building NICU at midday (panel A) and dawn/dusk (panel B). The lighting system luminaire is horizontally mounted above the patient headwall (insets). Intensity and spectral distribution vary from dawn through dusk. In panel B, additional overhead LED fixtures are shown in the inset picture. These provide additional lighting if needed for patient evaluation. All lights can be activated by bedside staff as necessary. The bars to the right of each panel demonstrate the research control interface which can be programed to generate variable spectra throughout the daylight period.

**Table 1:** 

Human Opsin	λ Max	Description
Rhodopsin (Rho)	496 nm	Expressed in retinal rod photoreceptors Low light vision
Short Wavelength-Sensitive Opsin (OPN1SW)	419 nm	Expressed in retinal cone photoreceptors Color vision
Medium Wavelength-Sensitive Opsin (OPN1MW)	531 nm	Expressed in retinal cone photorecpetors Color vision
Long Wavelength-Sensitive Opsin (OPN1LW)	558 nm	Expressed in retinal cone photoreceptors Color vision
Retinal G Protein-Coupled Receptor Opsin (RGR)	450 nm	Expressed in retina Chromophore regeneration
Peropsin (RRH)	Dark-activated	Expressed in retina Chromophore regeneration
Encephalopsin (0PN3)	430 nm	Non-visual opsin expressed in multiple extraocular tissues Energy homeostasis
Melanopsin (OPN4)	479 nm	Expressed in retinal ganglion cells Circadian entrain me nt of activity cycle
Neuropsin (OPN5)	380 nm	Non-visual opsin expressed in multiple extraocular tissues Energy homeostasis Peripheral tissue circadian clock entrainment

**Table 2:** 

Audience	Questions/Concerns	Answers	Tools Utilized for Dissemination
**Neonatologists and Bedside Care Staff**	What are the expected benefits of spectral lighting? Can light penetrate the skin? Is light detected only through the eyes? Are there any benefits to care staff? Are there wider implications for indoor lighting? Can this be used to benefit shift workers? Certain clinical conditions require a low stimulation environment. Can I turn off the lighting system based on my clinical judgement?	Spectral lighting could potentially help babies establish healthy wake/sleep cycles, regulate metabolism, promote optimal eye, and brain development and maintain a healthy body temperature Visible light variably penetrates the body No, Opsins are widely expressed throughout the body, including the skin Growing evidence indicates that spectral lighting may support better sleep patterns, improve alertness, regulate metabolism and mood. We see this research as an avenue to improve all indoor lighting environments Access to spectral lighting may benefit shift workers. More studies are needed to confirm this, Yes. The lighting system can be reactivated at the appropriate time and will follow the spectral composition corresponding to that time of day	Informational flyers in staff common areas Joint rounding on NICU unit with NICU nursing educator One on one conversations Small group conversations Brief (10 minute) presentations for specialized care teams Regularly scheduled communication and partnership with NICU educators Internal clinical digital forum (NICU Notes) CCHMC eCHirp (online educational resource for CCHMC NICU staff)
**Patient Families**	How does this help my child? Can the lights be turned off if 1 believe they are disturbing my child?	Spectral lighting could potentially help your baby gain weight and grow faster, sleep better, help their eyes and brain develop optimally and help maintain a healthy body temperature. Yes, please ask your child’s nurse or doctor.	Informational flyers provided at time of admission One on one conversations, informal rounding
